# Dynamic Schedule-Based Assignment Model for Urban Rail Transit Network with Capacity Constraints

**DOI:** 10.1155/2015/940815

**Published:** 2015-03-30

**Authors:** Baoming Han, Weiteng Zhou, Dewei Li, Haodong Yin

**Affiliations:** ^1^School of Traffic and Transportation, Beijing Jiaotong University, Beijing 100044, China; ^2^State Key Lab of Rail Traffic Control & Safety, Beijing Jiaotong University, Beijing 100044, China

## Abstract

There is a great need for estimation of passenger flow temporal and spatial distribution in urban rail transit network. The literature review indicates that passenger flow assignment models considering capacity constraints with overload delay factor for in-vehicle crowding are limited in schedule-based network. This paper proposes a stochastic user equilibrium model for solving the assignment problem in a schedule-based rail transit network with considering capacity constraint. As splitting the origin-destination demands into the developed schedule expanded network with time-space paths, the model transformed into a dynamic schedule-based assignment model. The stochastic user equilibrium conditions can be equivalent to the equilibrium passenger overload delay with crowding penalty in the transit network. The proposal model can estimate the path choice probability according to the equilibrium condition when passengers minimize their perceptive cost in a schedule-based network. Numerical example in Beijing urban rail transit (BURT) network is used to demonstrate the performance of the model and estimate the passenger flow temporal and spatial distribution more reasonably and dynamically with train capacity constraints.

## 1. Introduction

As Beijing urban rail transit (BURT) network is growing rapidly, the ridership of the BURT network is about 8,000,000 person-trips, which is still increased quickly successively. For safe and efficient operation requirements, the high risk of passenger flow and multistakeholders status makes it necessary and critical to estimate and evaluate the passenger flow temporal and spatial distribution scientifically and reasonably within the BURT network. With the automatic fare collection (AFC) [1] and passenger information system (PIS), the dynamic transit assignment problem, substantially, is a black-box problem with input of specific original-destination (O-D) information. Transit assignment model is a practical approach for estimating and predicting how passengers utilize transit system and choose paths, which can be definitely divided into two types: “frequency-based” and “schedule-based” [2]. On frequency-based transit network, each transit line is assumed to be run on a constant headway with a static process in the transit assignment [3–5], and the network would be represented in a static manner [6, 7]. For the majority of these models, schedule of transit system is assumed to be sufficiently reliable. Therefore, the headway is calculated by the average frequencies of transit line in frequency-based network. The waiting time and transfer time are implicit estimated based on headway. Since the time dimension is not considered in frequency-based transit model, the assignment results in frequency-based models are the average value in the specified time period (e.g., the rush hour). Unlike frequency-based type model, schedule-based models generally take explicitly timetable or schedule of the transit system into account [8–11], which means that the detailed departure or arrival times of vehicle or train in each transit lines are used in assignment procedures. According to different schedule-based transit assignment models, the time-dependent transit network representations can be classified into four types: (a) diachronic graph [12]; (b) dual graph [13]; (c) forward star network [14]; (d) discrete space-time graph [15] and time-expanded network [16]. Modeling formulations of transit assignment are one of schedule-based problems. Alfa and Chen [17] formulated a transit assignment model to forecast the temporal and spatial demand distribution in transit network. Tong and Wong [14] and Poon et al. [18] put forward a dynamic user equilibrium model, considering the crowded environment in boarding stations. Nielsen [19] proposed a stochastic transit assignment model considering differences properties in passengers' utility function. As capacity constraints considered in schedule-based network in transit assignment are gradually developed in recent years, Hamdouch and Lawphongpanich [16] developed the model of how passengers are unable to board vehicle due to capacity limited assigned in to waiting arcs of time expanded network. Nuzzolo et al. [20] presented a schedule-based dynamic assignment model with using joint choice model for transit network taking congestion into account through explicit vehicle capacity. Hamdouch et al. [21] and Sumalee et al. [22] considered one of the critical factors of capacity, sitting and standing capacities, and the treatment of seat allocation is considered as a random probability to get a seat or not. Since previous researches in passenger flow assignment models are limited in schedule-based urban rail transit network, especially in considering the particularity of rail transit system described above, and lack of considering train capacity, a new stochastic user equilibrium (SUE) rail transit assignment model is introduced and formulated to estimate the passenger flow temporal and spatial distribution within network during a given time interval.

Following the introduction, the remainder of this paper is organized as follows.  Section 2 focuses on network representation of the presented model. In Section 3, a SUE assignment model of SE network with trains' capacity constraint and the solution procedure is presented, while Section 4 illustrates the results of an application to the real test experiment. Conclusions of this study are reported in Section 5.

## 2. Network Representation

As the schedule diagrams the departure time from the first station and daily planned arrival or departure time at each station along the transit lines, this paper proposes a new method that represents the static network which incorporates the temporal information of the trains, called schedule-expanded network. Essentially, the SE network is expanded from the two-dimension route network *G*(*N*, *A*) with adding the time dimension. The SE network of BURT network is of the form *G*(*N*, *A*, *T*), where **N** denotes the sets of stations, **A** denotes the arcs of the sections of lines, and **T** denotes sets of the scheduled arrival/departure time of trains. The basic elements of the SE network are described as follows.

*(1) Temporal Nodes.* Let *r*_*t*_^*l*^ denote the temporal node, where the trains run through station **r** of line **l** at time **t**. In general, if there are *n* lines crossing the station and *k* trains running on each line, the station will be expanded into *n*∗*k* temporal nodes.

*(2) Temporal Arcs.* Let *a*(*r*_*t*_1__^*l*^, *s*_*t*_2__^*l*^) denote the temporal section arcs from temporal node *r*_*t*_1__^*l*^ to temporal node *s*_*t*_2__^*l*^of line *l*. Similarly an arc **a**(**r**, **s**) of route network is expanded into *k* arcs as the form (*r*_*t*_1__^*l*^, *s*_*t*_2__^*l*^)_*k*_ where *k* equals the number of trains running though the section of the line based on schedule. As defined in the previous section, *c*_*rs*_ denotes the travel time of arc (*r*, *s*). Let *c*_*t*_1_,*t*_2__^*rs*,*l*^ denote the travel time of temporal section arc *a*(*r*_*t*_1__^*l*^, *s*_*t*_2__^*l*^), and let *t*_1_, *t*_2_ be the arrival time at nodes *r* and *s*. *c*_*t*_1_,*t*_2__^*rs*,*l*^ can be calculate as follows:

(1) $$\begin{array}{c} c_{t_{1},t_{2}}^{rs}=t_{2}-t_{1}\;t_{1},t_{2}\in T,\;\;r,s\in N,\;\;a\in A. \end{array}$$

Let *a*(*r*_*t*_1__^*l*_1_^, *r*_*t*_2__^*l*_2_^) denote the temporal transfer arcs of the station **r** from line *l*_1_ to *l*_2_. As the endpoints of the arcs are the temporal nodes *r*_*t*_1__^*l*_1_^ and *r*_*t*_2__^*l*_2_^, so the travel time *c*_*t*_1_′,*t*_2_′_^*r*,*l*_1_*l*_2_^ of the transfer arc (*r*_*t*_1__^*l*_1_^, *r*_*t*_2__^*l*_2_^) is

(2) $$\begin{array}{c} c_{t_{1}^{\prime },t_{2}^{\prime }}^{r,l_{1}l_{2}}=t_{2}^{\prime }-t_{1}^{\prime }\;t_{1}^{\prime },t_{2}^{\prime }\in T,\;\;r,s\in N,\;\;a\in A. \end{array}$$

In addition, there are temporal arcs (*r*_*t*_1__, *r*_*t*_2__) that represent passengers having to wait at station **r** from time *t*_1_ to *t*_2_, defined as *a*(*r*_*t*_1__^*l*^, *r*_*t*_2__^*l*^). Let $c_{{}_{\bar{t_{1}},t_{2}}}^{r,l}$ denote the passengers waiting time in temporal nodes *r*_*t*_1__^*l*^ at the arrival time $\bar{t_{1}}$ and leave at scheduled departure time *t*_2_:

(3) $$\begin{array}{c} c_{{\;}_{\bar{t_{1}},t_{2}}}^{r,l}=t_{2}-\bar{t_{1}}\;\bar{t_{1}},t_{2}\in T,\;\;r,s\in N,\;\;a\in A. \end{array}$$

**Figure 1:**
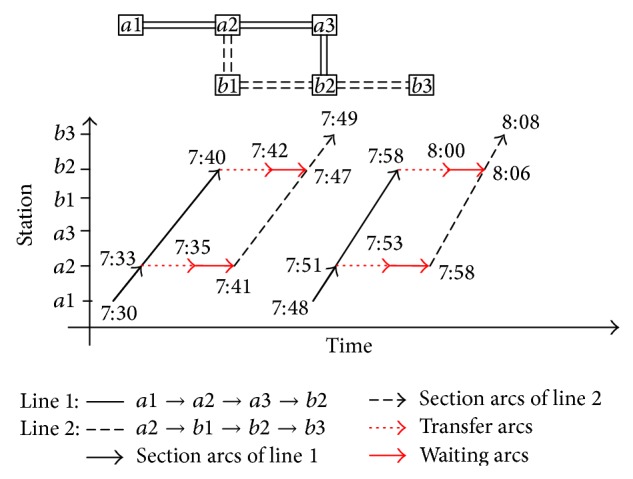
A SE network based on the route network of one O-D pair in time period.

Figure 1 shows the SE network based on the route network upside, and the scheduled times of Figure 1 are shown in Table 1. Actually, Figure 1 shows all possible paths from station *a*1 to *b*3 at 7:30 to 8:10 a.m. For example, the path from *a*1_7:30_^*L*1^ → *a*2_7:33_^*L*1^ → *a*2_7:41_^*L*2^ → *b*3_7:49_^*L*2^ corresponds to passengers leaving node *a*1 at time 7:30 with train 0L11, arriving at *a*2 of Line 1 at 7:33, then transferring to *a*2 of Line 2 at 7:42, waiting for 0L21 and boarding train 0L21 at 7:47, and finally reaching *b*3 at 7:49.

In the SE network, temporal paths of O-D stations are a sequence of temporal arcs. Generally, a temporal path always consists of the 3 kinds of arcs proposed above. Computationally, schedule-expanded nodes and arcs can often be generated using the route network and timetable when solving an optimization problem, for example, to find the most optimal hyperpaths, the least travel time cost, and the most optimal path between O-D pairs. As the possible temporal paths consist of hyperpath with time dimension, *k*-shortest paths searching algorithm [6, 14] can be used in searching the possible temporal paths, if and only if the condition holds:

(4) $$\begin{array}{c} t_{k}^{o_{i}d_{j}}\leq \min\left\{t_{k^{\prime }}^{o_{i}d_{j}}+t_{\text{tor}},t_{k^{\prime }}^{o_{i}d_{j}}*\left(1+d1\right)\right\},\\ t_{k}^{\text{wat},o_{i}d_{j}}\leq \min\left\{t_{k^{\prime }}^{\text{wat},o_{i}d_{j}}+t_{\text{wat},\text{tor}},t_{k^{\prime }}^{\text{wat},o_{i}d_{j}}*\left(1+d2\right)\right\},\\ t_{k}^{n}\leq N, \end{array}$$

where *t*_*k*_^*o*_*i*_*d*_*j*_^ denotes the travel time of *k*th path *l*_*k*_ in path set *L*_*t*_list__, *t*_*k*′_^*o*_*i*_*d*_*j*_^ denotes the minimum travel time of the path set *L*_*t*_list__, *t*_*k*′_^wat,*o*_*i*_*d*_*j*_^ denotes the minimum waiting time of the path set, *t*_tor_ denotes the maximum tolerance travel time of the path, *t*_wat,tor_ denotes the maximum tolerance waiting time of the path, *t*_*k*_^*n*^ denotes the number of transfers times, *N* denotes the maximum tolerance number of transfers times, and *d*1 and *d*2 denote the maximum magnification of the tolerance time. All these tolerance parameters are user-defined.

## 3. Stochastic Dynamic User Equilibrium Assignment Model

### 3.1. Generalized Travel Time Cost in SE Network

The generalized travel time cost *gc* ^*rs*^ on arc (*r*, *s*) is subjected to a disutility that encompasses four weighted combination components: (i) in-vehicle travel time cost *gc*_ivt_^*rs*^; (ii) waiting time cost *gc*_WAT_^*r*,*l*^; (iii) transfer time cost *gc*_trt_^*r*,*l*_1_*l*_2_^; (iv) passenger overload cost $g{\tilde{c}}_{{}_{t_{1},t_{2}}}^{r,l}$. Denote *β*_1_, *β*_2_ by the weighting factors for waiting time and transfer time, respectively, and *β*_3_ by the passenger overload delay in weighted time unit parameter. The generalized travel time cost *gc* ^*rs*^ of the arc *a*(*r*, *s*) can be expressed as

(5) $$\begin{array}{c} gc^{rs}=gc_{\text{ivt}}^{rs}+gc_{\text{WAT}}^{r,l}+gc_{\text{trt}}^{r,l_{1}l_{2}}+g{\tilde{c}}_{{}_{t_{1},t_{2}}}^{r,l}. \end{array}$$

Considering the components of the generalized travel time cost function in SE network, the components on the right side of the equation in a time interval Δ*t* may be described as follows.

(i) If the number of passengers is large enough to make passengers discomfortable, the cost of the in-vehicle travel time, as *T*_IVT_^*rs*^, may be amplified. When the number of the passengers is small, for example, less than the number of seats, it is not crowded in the train. Let *ω* = *x*_*t*_1_,*t*_2__^*rs*^/*C*_*l*_ denote trainload. With the trainload increasing, passengers may feel more discomfortable. We defined a piecewise function to describe the discomfort level with the passengers number increasing and the trainload in the temporal section arcs as

(6) $$\begin{array}{c} f_{c}\left(x_{t_{1},t_{2}}^{rs}\right)=\left\{\begin{array}{cc} 1;\;\frac{x_{t_{1},t_{2}}^{rs}}{C_{l}}\leq \omega_{0}, & \\ 1+\phi_{1}\left(x_{t_{1},t_{2}}^{rs}\right)*\left(\frac{x_{t_{1},t_{2}}^{rs}}{C_{l}}-\omega_{0}\right), & \\ \;\;\omega_{0}<\frac{x_{t_{1},t_{2}}^{rs}}{C_{l}}\leq \omega_{1}; & \\ 1+\phi_{1}\left(\omega_{1}*C_{l}\right)*\left(\omega_{1}-\omega_{0}\right) & \\ \;+\;\phi_{1}\left(x_{t_{1},t_{2}}^{rs}\right)*\left(\frac{x_{t_{1},t_{2}}^{rs}}{C_{l}}-\omega_{1}\right); & \\ \;\;\omega_{1}<\frac{x_{t_{1},t_{2}}^{rs}}{C_{l}}; & \end{array}\right.\\ \omega_{0}=\frac{Z_{l}}{C_{l}},\;\phi_{1}\left(x_{t_{1},t_{2}}^{rs}\right)=\alpha_{1}*{\left(\omega \right)}^{\gamma },\;\omega =\frac{x_{t_{1},t_{2}}^{rs}}{C_{l}}, \end{array}$$

where *Z*_*l*_ denotes the seat number in the train and *α*_1_ and *γ* are user-defined factors. The in-vehicle travel time cost in SE network is

(7) $$\begin{array}{c} gc_{\text{ivt}}^{rs}=f_{c}\left(x^{rs}\right)*T_{\text{IVT}}^{rs}=c_{t_{1},t_{2}}^{rs}*f_{c}\left(x_{t_{1},t_{2}}^{rs}\right). \end{array}$$

(ii) According to train schedule, it is applicable to calculate the waiting time *T*_WAT_^*rs*^ by the subscripts of the temporal arc:

(8) $$\begin{array}{c} gc_{\text{WAT}}^{r,l}=\beta_{1}*T_{\text{WAT}}^{rs}=\beta_{1}*c_{{}_{t_{1},t_{2}}}^{r,l}. \end{array}$$

(iii) Transfer time includes two factors: the transfer times and the walking time *T*_TRT_^*rs*^ from one line transfer to the other line at a transfer station. That is because the transfer contains the process of “alighting-walking-boarding.” So the passengers have to change from one vehicle/train to another vehicle/line which will increase the passengers' extra perceived costs. Letting *σ* denote the extra perceived cost factor which is affect by the transfer times, transfer time cost can be expressed as

(9) $$\begin{array}{c} gc_{\text{trt}}^{r,l_{1}l_{2}}=\beta_{2}*T_{\text{TRT}}^{rs}+\sigma =\beta_{2}*c_{t_{1},t_{2}}^{r,l_{1}l_{2}}+\sigma . \end{array}$$

(iv) Passenger overload delay is the additional time that passengers spend on waiting for next train of temporal section arc due to the insufficient train capacity when they cannot board the first coming train of the temporal section. Let *β*_3_ denote the passenger overload delay penalty factor and let *m*_*rs*,*k*_ denote the overload delay of temporal arc (*r*, *s*) in path *k*:

(10) $$\begin{array}{c} g{\tilde{c}}_{t_{1},t_{2}}^{r,l}=\beta_{3}*m_{rs,k}. \end{array}$$

Combined with the generalized travel time cost *gc* ^*rs*^ of the temporal arc *a*(*r*, *s*) in temporal arcs list, the generalized travel time cost to reach at destination *d*_*j*_ from original *o*_*i*_ node *GC*_*k*_^*o*_*i*_*d*_*j*_^ can be expressed as

(11) $$\begin{array}{c} gc^{rs}\left(x_{t_{1},t_{2}}^{rs}\right)=gc_{\text{ivt}}^{rs}+gc_{\text{wat}}^{r,l}+gc_{\text{trt}}^{r,l_{1}l_{2}}+g{\tilde{c}}_{\text{ody}}^{r,l},\;a\left(r,s\right)\in a,\\ GC_{k}^{o_{i}d_{j}}=\underset{(r,s)\in a}{\sum }gc_{t_{1},t_{2}}^{rs}*\delta_{rs,k}^{o_{i}d_{j}},\\ GC_{k}^{o_{i}d_{j}}=\infty ,\;\text{if}\;\;t\notin \Delta t. \end{array}$$

### 3.2. Flow Conservation in SE Network

Passenger flows on temporal arcs which satisfy the following constraints. For each O-D pair *o*_*i*_ *d*_*j*_, the trip demand *q* ^*o*_*i*_*d*_*j*_^ can be split into all effective temporal paths as

(12) $$\begin{array}{c} q^{o_{i}d_{j}}=\underset{l_{k}\in L_{t_{\text{list}}}}{\sum }v_{k}^{o_{i}d_{j}}, \end{array}$$

where *v*_*k*_^*o*_*i*_*d*_*j*_^ denotes the passenger flows assign to the path *l*_*k*_ of each *o*_*i*_ *d*_*j*_.

Each temporal arc *a*(*r*, *s*) should satisfy the flow conservation of each specific temporal path as

(13) $$\begin{array}{c} v^{rs}=\underset{a(r,s)\in a\;\;}{\sum }\underset{l_{k}\in L_{t_{\text{list}}}}{\sum }\delta_{rs,k}^{o_{i}d_{j}}v_{k}^{o_{i}d_{j}}, \end{array}$$

where *v* ^*rs*^ denotes the passenger flows assign to *a*(*r*, *s*).

Furthermore, temporal arcs flow should satisfy the capacity constraint where would not be an overload in the rail transit train to which the temporal section arcs *a*(*r*, *s*) belong:

(14) $$\begin{array}{c} v^{rs}\leq K^{rs,l}, \end{array}$$

where *K* ^*rs*,*l*^ denotes the capacity *a*(*r*, *s*) of in line **l**.

### 3.3. SUE Assignment Model Formulation

By using the schedule expanded network, the temporal and spatial variation of passengers' trip would be represented by the temporal path in network. The temporal paths in the network are associated with time and space. Hence, the dynamic rail transit assignment can be transformed into static transit assignment problem.

According to the definition, a stochastic user equilibrium (SUE) is achieved in a schedule-based transit network when the allocation of passengers between alternative paths conforms to the following logit model:

(15) $$\begin{array}{c} \mathrm{In}\left(\frac{f_{k}^{o_{i}d_{j}}}{f_{k^{\prime }}^{o_{i}d_{j}}}\right)=-\theta \left(GC_{k}^{o_{i}d_{j}}-GC_{k^{\prime }}^{o_{i}d_{j}}\right), \end{array}$$

where *k* and *k*′ are the alternative temporal paths *l*_*k*_ and *l*_*k*′_ associated with the same O-D pair *o*_*i*_ *d*_*j*_ and *θ* > 0 is a given parameter which is used to measure the different degree of passengers' knowledge about state of the paths in the network. The parameter *θ* will increase when passengers are more familiar with the schedule and travel time cost of the network. As *θ* → *∞*, the SUE approximately is equal to that of user equilibrium (UE).

Based on (11) and (15), we have

(16) In⁡fkoidjfk′oidj =−θ(gct1,t2k−gct1,t2k′)+(gct1,t2k,l−gct1,t2k′,l)    +gct1,t2k,l1l2−gct1,t2k′,l1l2+gc~t1,t2k,l−gc~t1,t2k′,l.

As the total travel demands increase, the proportionate distribution of passenger flow between the two paths remains the same until one or more temporal arcs on either path are saturated. If *v* ^*rs*^ = *K* ^*rs*,*l*^, further increase in total travel demands would cause congestion on paths, leading to the increase overload delay, which will affect the path choice. And the crowding penalty and the overload delay are the equilibrium mechanism of logit-based assignment model in this paper.

The SUE assignment problem of the SE network will be formulated as follows:

(17) P1:       min⁡ Z=∑(r,s)∈a∫0vrsgcrs(x)dx +1θ∑ar,s∈k  ∑k∈Llistvkoidj∗In⁡vkoidj,

(18)     s.t.  qoidj=∑k∈Ltlistvkoidj,

(19)       vrs=∑ar,s∈a  ∑k∈Ltlistδrs,koidjvkoidj,

(20)       vrs≤Krs,l,

(21)       vkoidj≥0,

(22)       ∀ar,s∈a, k∈Ltlist.

The equivalence and uniqueness of the model have been proved in study (6). By constructing the* Lagrangian* function for problem** P1**, the* Kuhn-Tucker* conditions of** P1** can be given as follows:

(23) $$\begin{array}{c} \mathrm{In}v_{k}^{o_{i}d_{j}}+\theta *\underset{\left(r,s\right)\in a}{\sum }(gc_{}^{rs}*\delta_{rs,k}^{o_{i}d_{j}}-m_{rs,k}^{o_{i}d_{j}})-l_{o_{i}d_{j}}=0, \end{array}$$

where *m*_*rs*,*k*_^*o*_*i*_*d*_*j*_^ and *l*_*o*_*i*_*d*_*j*__ are the corresponding* Lagrangian* multiplier to (19) and (20). Equation (17) can be easily transformed into the following logit-based model, which is the *k*th path probability of path *l*_*k*_ in *L*_*t*_list__:

(24) $$\begin{array}{c} p_{k}^{o_{i}d_{j}}=\frac{\exp\left(-\theta GC_{k}^{o_{i}d_{j}}+m_{k}^{o_{i}d_{j}}\right)}{\sum_{i\in L_{t_{\text{list}}}}\exp\left(-\theta GC_{i}^{o_{i}d_{j}}+m_{k}^{o_{i}d_{j}}\right)}, \end{array}$$

where *m*_*k*_^*o*_*i*_*d*_*j*_^ denotes the sum of* Lagrangian* multipliers *m*_*rs*,*k*_^*o*_*i*_*d*_*j*_^ along path *l*_*k*_ in *L*_*t*_list__.

### 3.4. Solution Procedures

If the capacity constraints are ignored, problem** P1** becomes a standard SUE assignment on transit network. This paper developed an advanced method of successive averages (MSA) algorithm to solve problem** P1** with capacity constraints.

Rewrite (23) as

(25) $$\begin{array}{c} v_{k}^{o_{i}d_{j}}=\mathrm{In}\left(-\theta \left(gc_{\text{ivt}}^{rs}+gc_{\text{wat}}^{r,l}+gc_{\text{trt}}^{r,l_{1}l_{2}}\right)\right)\underset{a(r,s)\in k}{\prod }M_{rs}L_{\text{OD}}, \end{array}$$

where *L*_OD_ = exp⁡(*l*_*o*_*i*_*d*_*j*__) and *M*_*rs*_ = exp⁡(*m*_*rs*,*k*_^*o*_*i*_*d*_*j*_^). *M*_*rs*_ is the factor corresponding to overload delay and is to be determined at the arc *a*(*r*, *s*) ∈ *l*_*k*_. With the advanced MSA, a simple solution procedure is put forward to solve the problem** P1** with the given O-D demands *q* ^*o*_*i*_*d*_*j*_^ within time interval Δ*t*.

**Step 1 (network construction).**

Calculate the temporal possible paths set *L*_list_ in the analytical network with SE network representation method for all O-D pairs *o*_*i*_ *d*_*j*_ ∈ OD.

**Step 2 (initialization).**

Set *L*_OD_^*n*^ = 1 and *M*_*rs*_^*n*^ = 1 for each arc *a*(*r*, *s*) ∈ *l*_*k*_ for ∀*o*_*i*_ *d*_*j*_ ∈ OD, and set iterations *n* = 1.

**Step 3 (iteration).**

Calculate ((26)-(27)) step by step until the convergent conditions are satisfied for each *a*(*r*, *s*) ∈ *k* and *o*_*i*_ *d*_*j*_ ∈ OD.

For each *a*(*r*, *s*) ∈ *k*, calculate the following equation:

(26) vkoidj(L→n,M→n) =exp⁡(−θ(gcivtrs+gcwatr,l+gctrtr,l1l2))∏a(r,s)∈kMrsnLoidjn,ηrsn=Kt1,t2rs,l∑oidj∈OD∑k∈Ltlistδrs,koidjvkoidjL→n,M→n,Mrsn+1=min⁡1,ηrsnMrsn.

For each *o*_*i*_ *d*_*j*_ ∈ OD, calculate the following equation:

(27) $$\begin{array}{c} \eta_{o_{i}d_{j}}^{n}=\frac{q^{o_{i}d_{j}}}{\sum_{k\in L_{\text{list}}}b_{o_{i}d_{j},k}v_{k}^{o_{i}d_{j}}\left({\vec{L}}^{n},{\vec{M}}^{n}\right)},\\ L_{o_{i}d_{j}}^{n+1}=\eta_{o_{i}d_{j}}^{n}L_{o_{i}d_{j}}^{n}. \end{array}$$

Then, *n* = *n* + 1.

Let ${\vec{L}}^{n}$ and ${\vec{M}}^{n}$ be the sets of *l*_*o*_*i*_*d*_*j*__ and *m*_*rs*,*k*_^*o*_*i*_*d*_*j*_^. The superscript *n* denotes iterations, *b*_*o*_*i*_*d*_*j*_,*k*_ denotes arc-demand incidence factor equal 1 if $v_{k}^{o_{i}d_{j}}({\vec{L}}^{n},{\vec{M}}^{n})$ loading in path *l*_*k*_ belongs to O-D demands *q* ^*o*_*i*_*d*_*j*_^, else *b*_*o*_*i*_*d*_*j*_,*k*_ = 0.

**Step 4 (convergence and output results).**

Calculate the gap function, if $G(n)=\sqrt{\sum_{o_{i}d_{j}\in \text{OD}}\delta_{rs,k}^{o_{i}d_{j}}{\left(v_{k}^{o_{i}d_{j},n+1}-v_{k}^{o_{i}d_{j},n}\right)}^{2}}/\sum_{o_{i}d_{j}\in \text{OD}}q^{o_{i}d_{j}}<\varepsilon$, then stop and *ε* is the presupposed gap factor. Then, output arcs flow and overload delay value.

For each *a*(*r*, *s*) ∈ *k*, calculate

(28) $$\begin{array}{c} v^{rs}=\underset{a(r,s)\in a\;\;}{\sum }\underset{k\in L_{t_{\text{list}}}}{\sum }\delta_{rs,k}^{o_{i}d_{j}}v_{k}^{o_{i}d_{j},n}\left({\vec{L}}^{n},{\vec{M}}^{n}\right),\\ m_{rs,k}^{o_{i}d_{j}}=-\frac{\left(\ln M_{rs}^{n}\right)}{\theta \beta_{3}}. \end{array}$$

Else, back to Step 3.

## 4. Numerical Example of Model Application in BURT Network

**Figure 2:**
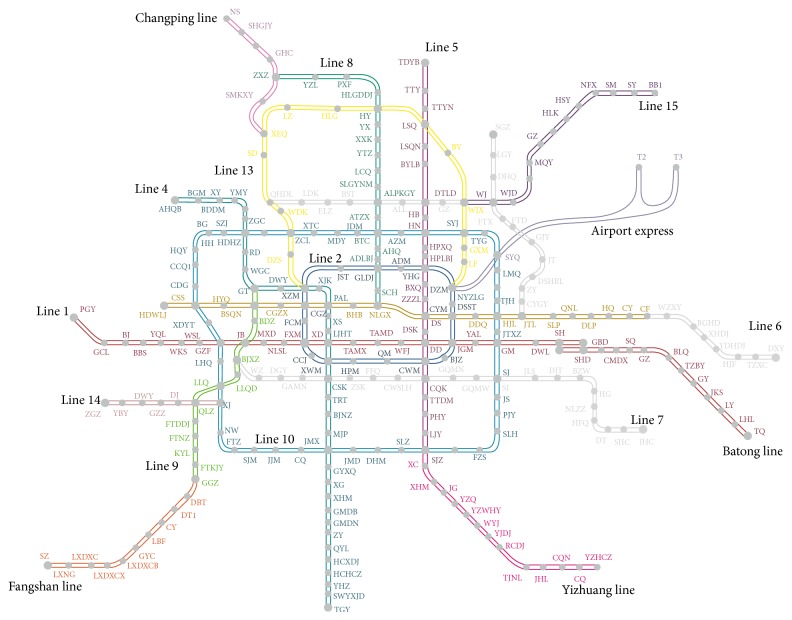
Beijing urban rail transit network (April, 2014).

The proposed models and solution algorithm are used to apply to Beijing urban railway transit network for passenger flow distribution estimation. The network consists of 17 lines and 281 stations including 41 transfer stations, which is shown in Figure 2. There are a total of 562 nodes, 624 running sections, and 90 transfer arcs in the topological network.

In this study, the input data of the assignment model requires the train running schedules of each transit line, the transfer walking time of each transfer, and the train capacity of each line. Total O-D demands of this time period (7:00~9:00 a.m.) are 1095835 among 54468 O-D pairs. Parts of these data are from the website of Beijing Metro Company, Wikipedia of Beijing Subway, and Beijing Municipal Commission of Transport (BMCT); others are from the corresponding surveys. Parts of the input data are shown in Table 2. Since the schedule data is a large table with over 40,000 records in data table, Table 2 only shows partial data.

In order to verify the results of the** P1** above, we select one day in April 2014 of the AFC system to obtain the O-D demands as presented above which is provided from the BMCT.

Parameters are calibrated by SPSS statistically with the existing research data, which is shown in Table 3. The assignment period is from 7:00 a.m. to 9:00 a.m. The network assignment is running on an AMD Core 16 quad, 8 Gb Ram server. The computing time is about 20 minutes.

**Figure 3:**
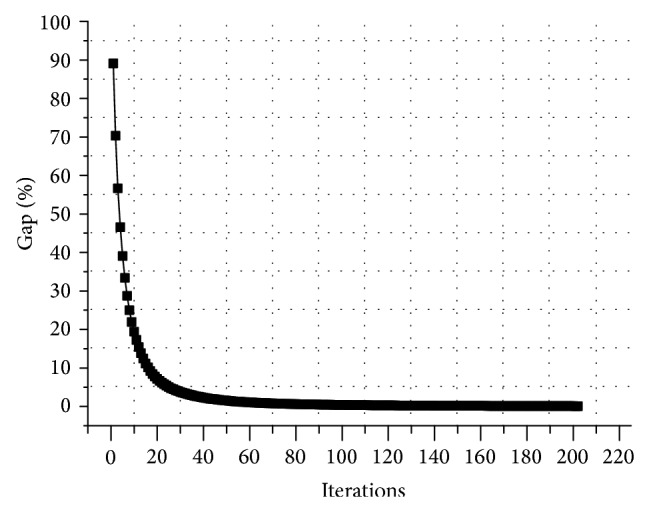
Convergence characteristics for *θ* = 1.

Figure 3 indicates the iterations of the MSA for *θ* = 1. The gap function converges rapidly at the beginning and slowly after 80 iterations (0.637%), suggesting that the advanced MSA has good convergence property in large scale network calculation. In this study, the presupposed gap factor *ε* = 0.1%. The final gap function is 0.1%, which indicates that the assignment results are much close to the equilibrium results. Seeing that the gap function is less than 0.5% after iterating 92 times (0.49%) which is demonstrated in Figure 3, solution results with less than 0.5% gap function are expected to be acceptable in practice, which the iterations and calculating time can be reduced.

**Figure 4:**
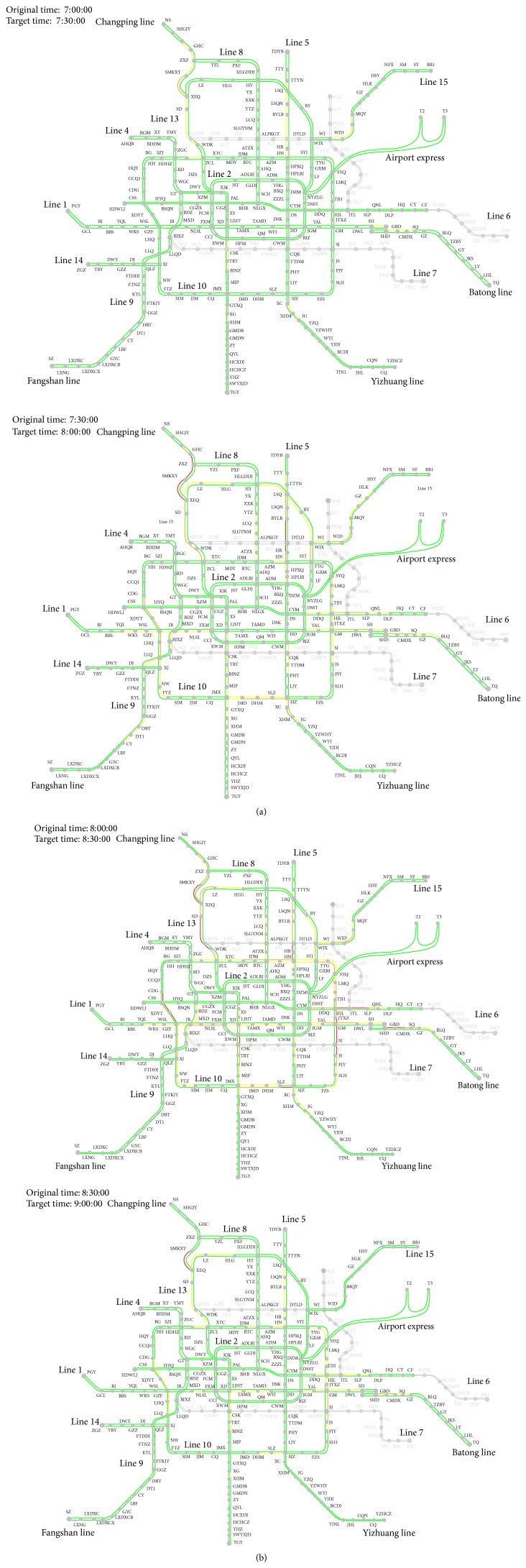
Passenger flow distribution in BURT network 7:00–9:00 a.m., each figure displays one time interval of 30 minutes.

The computation results are showed in Figure 4 from 7:00 a.m. to 9:00 a.m., and the time interval is 30 minutes. In order to illustrate assignment results of passengers flow distribution in BURT network directly, this paper develops a system which outputs trainload of each section. Besides, this paper defines different color of the transit line sections to indicate the trainload which is shown in Figure 4. The colors are defined to be four degrees of trainload, where green indicates *ω* ≤ 0.8, yellow indicates 0.8 < *ω* ≤ 1, orange indicates 1 < *ω* ≤ 1.2, and red indicates *ω* > 1.2.

**Figure 5:**
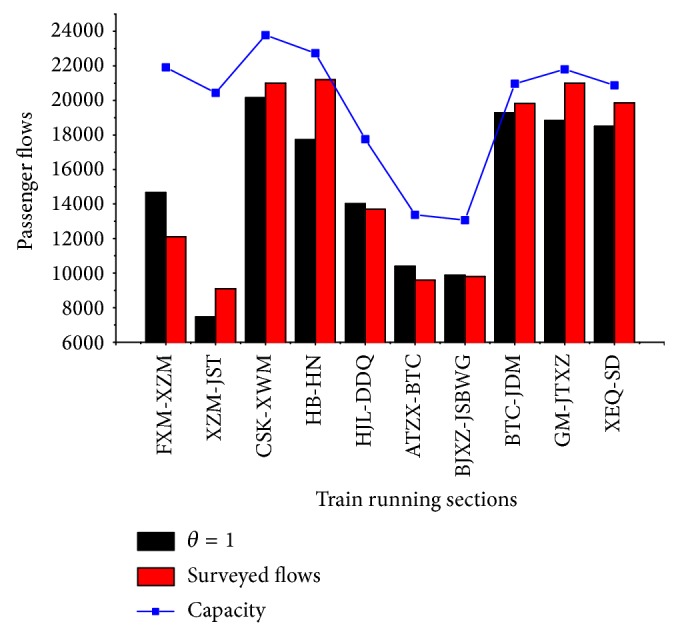
Comparison of surveyed flows, computed flows, and capacity in transit sections.

To further analyze the computing results, we compare the computing flows distributing on sections of the lines with the survey data, which includes passenger flows and trainload during the same section in Figure 5. It can be obviously seen that the average errors between computing and observed passenger flows are acceptable.

**Figure 6:**
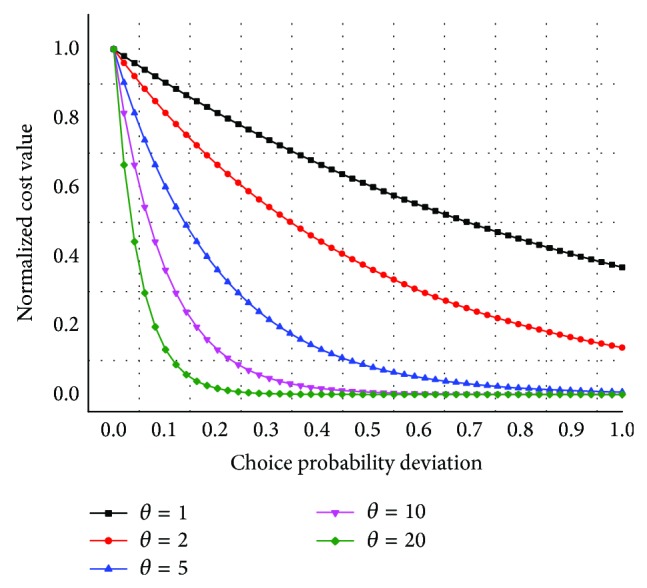
Path choice probability deviation with different values of *θ*.

**Figure 7:**
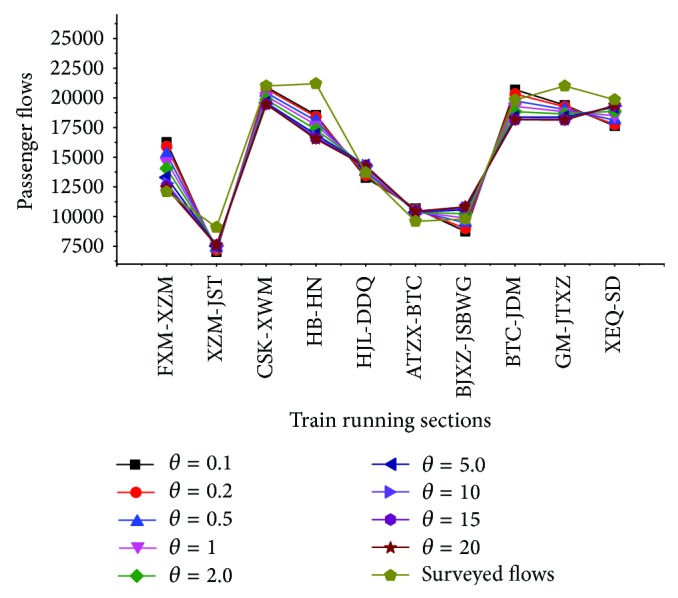
Impacts of *θ* in assignment results of train running sections.

In addition, the parameter sensitivity test can be put forward to obtain full understanding of the proposal model. This study changes the parameter *θ* at a time with all the other parameters being fixed to estimate different assignment results by the assignment method.  Figure 6 indicates the path choice probability deviation when taking different values of *θ*. Some resultant sections over delays are shown in Table 4 of the whole network for *θ* = 0.1 to 20, and flows loading on some sections are shown in Figure 7 when *θ* takes different values.

(1) The vertical axis in Figure 6 represents the normalized value comparison to the path with the least generalized travel time cost. The horizontal axis in Figure 6 represents the choice probability deviation. A large value of *θ* means that passengers are more sensitive to the difference between the generalized travel costs of their paths. When the value of *θ* increases, subtle difference in the generalized cost between the alternative paths will lead to a huge probability deviation in the choice probability. Hence, the path choice probability deviation distribution curve is smooth with a small value of *θ* and steep with a large value of *θ*.

(2) It can be seen in Table 4 that the overload delay of the whole network and the partial network would be increased with the increase of parameter *θ*. With the increase of parameter *θ*, the passengers would have more knowledge of the network with the paths, congestion status, and schedule or timetable in choosing the departure time and trains of temporal paths. As there is no other path with less generalized travel time cost, passengers would like to board the first coming train until full, which will increase congestion cost because passengers understand clearly that waiting for the next train may not reduce the total cost. And the assignment results have demonstrated the phenomenon that since each passenger has high degree of knowledge for network, they tend to choose the same path as they expected at the same time; however, this increases actual overload delay cost because of the capacity constraint.

(3) As the parameter *θ* varies from 0.1 to 20, it can be seen that the assignment results change slightly in Figure 6 during 8:00 to 8:30 a.m. As presented above, a large value of *θ* means a full knowledge of the network, with a less perception generalized travel time cost function error, and the passengers tend to choose the optimal path with minimum perception cost. A small value of *θ* indicates that passengers would choose many paths including some high cost paths at the beginning of their travel randomly due to the limited understanding of the network. As the assignment results in different values of the parameter *θ* comparing with the surveyed flows, the value of *θ* is better to take the range of [1,5] while the average error is 10.1%~12.3% to the surveyed flows.

## 5. Conclusion

A SUE assignment model with capacity constraints has been described in this paper to estimate and predict the passenger flow temporal and spatial distribution in the rail transit network. An advanced MSA algorithm is presented to solute the assignment model which is incorporated with the stochastic effect of passengers' choice behaviors, the schedule of transit system, capacity constraints of trains, and a large-scale rail transit network. By using schedule expanded network representation, the time-space dynamic assignment problem is formulated as a generalized cost model. Furthermore, this study considers some important factors of passengers, for example, passenger overload delay, which they determine to minimize in their path choice. Passenger overload delay is defined as unknown variables which are determined by the equilibrium mechanism and train capacity constraints with the arcs generalized cost function used in this study. Also this paper analyzes the equilibrium mechanism equivalent to the condition that the mathematical problem will be solved to the equilibrium passenger overload delay when the arcs reach their capacity level.

The model provides an important idea to evaluate the performance of the rail transit system subjected to train running sections load. The numerical example in application of BURT network in this study demonstrates that this model can be used on practical large-scale network due to the rapid convergence (within 200 iterations) and reasonable precision (10.1%~12.3%) in practice. With further research of the parameter sensitivity, the results indicate that passengers with better knowledge of the network would result in more overload delay in their trips and cause various assignment results.

As BURT network expanded rapidly, it has become a more and more critical task to scientifically estimate the passenger flow distribution in the network. For further research, the proposal model applied to BURT network will be extended with passenger behaviors, such as queuing choice and transfer station choice.

## Figures and Tables

**Table 1:** Partial schedule of the small network.

Train number	Station	Arrival time	Departure time	Line	Direction
0L11	a1	—	7:30	Line 1	Upstream
0L11	a2	7:33	7:34	Line 1	Upstream
0L11	b2	7:40	—	Line 1	Upstream
0L13	a1	—	7:48	Line 1	Upstream
0L13	a2	7:51	7:52	Line 1	Upstream
0L13	b2	7:58	—	Line 1	Upstream
0L21	A2	—	7:41	Line 2	Upstream
0L21	B2	7:46	7:47	Line 2	Upstream
0L21	B3	7:49	—	Line 2	Upstream
0L23	A2	—	7:58	Line 2	Upstream
0L23	B2	8:05	8:06	Line 2	Upstream
0L23	B3	8:08	—	Line 2	Upstream

**(a):** 

Train running schedule of transit lines (part of the data)					
Train number	Station	Arrival time	Departure time	Line	Direction
410000	Fuxingmen	7:03:43	7:04:28	Line 1	Upstream
410002	Fuxingmen	7:05:48	7:06:33	Line 1	Upstream
410001	Fuxingmen	7:01:12	7:01:47	Line 1	Downstream
410003	Fuxingmen	7:03:17	7:04:02	Line 1	Downstream
420000	Xizhimen	7:00:15	7:01:15	Line 2	Upstream
420002	Xizhimen	7:02:15	7:03:15	Line 2	Upstream
420001	Xizhimen	7:01:34	7:02:34	Line 2	Downstream
420003	Xizhimen	7:03:34	7:04:34	Line 2	Downstream
⋮					

**(b):** 

Transfer walking time at different transfer station (parts of the data)							
Transfer stations	Original line	Terminal line	Transfer walking time (/s)	Transfer stations	Original line	Terminal line	Transfer walking time (/s)

Xizhimen	Line 2	Line 4	164	Fuxingmen	Line 1	Line 2	394
	Line 4	Line 2	209		Line 2	Line 1	150

Dongzhimen	Line 2	Line 13	358	Jianguomen	Line 1	Line 2	169
	Line 13	Line 2	270		Line 2	Line 1	81

Xidan	Line 1	Line 4	235	Dongdan	Line 1	Line 5	275
	Line 4	Line 1	360		Line 5	Line 1	323

Zhichunlu	Line 10	Line 13	241	Dongsi	Line 5	Line 6	357
	Line 13	Line 10	225		Line 6	Line 5	339

National Library	Line 4	Line 9	166	Xuanwumen	Line 2	Line 4	166
	Line 9	Line 4	45		Line 4	Line 2	619

Beitucheng	Line 8	Line 10	85	Guomao	Line 1	Line 10	309
	Line 10	Line 8	90		Line 10	Line 1	322

Huoying	Line 8	Line 13	315	Shaoyaoju	Line 10	Line 13	211
	Line 13	Line 8	215		Line 13	Line 10	207

Chongwenmen	Line 2	Line 5	238	Liuliqiao	Line 9	Line 10	80
	Line 5	Line 2	392		Line 10	Line 9	103

Hujialou	Line 6	Line 10	142	Lishuiqiao	Line 5	Line 13	217
	Line 10	Line 6	127		Line 13	Line 5	231

**(c):** 

Train capacity of each line (persons per vehicle)						

Lines	Line 1	Line 2	Line 4	Line 5	Line 6	Line 8
Capacity	1850	1850	1830	1840	2570	1980
Lines	Line 9	Line 10	Line 13	Line 14	Line 15	
Capacity	1900	1900	1850	2570	1980	
Lines	Changping line	Yizhuang line	Fangshan line	Daxing line	Batong Line	Airport express
Capacity	1900	1900	1900	1830	1900	448

**Table 3:** The values of the parameter in the model.

Parameters	*β*_1_	*β*_2_	*β*_3_	*ε*	*α*_1_	*γ*
Values	1.5	2	1	0.1%	0.15	4
Parameters	*σ*	*t*_tor_	*t*_wat,tor_	*d*1	*d*2	*N*
Values	3.0	20	15	10%	5%	3

**Table 4:** Overload delays of transit sections for different values of the parameter *θ*.

Transit sections	Line	Overload delays of transit sections for different values of *θ* (/s)							
		0.1	0.2	0.5	1	2	5	10	20
FXM-XD	1	74.25	83.55	89.01	106.25	113.83	119.02	137.15	151.38
ZSM-JST	2	15.07	18.92	22.12	24.38	25.72	26.84	28.43	34.53
CSK-XWM	4	22.22	28.21	31.90	34.61	38.12	41.73	43.03	46.84
HB-HN	5	150.61	165.12	172.53	178.7	189.3	197.8	201.3	206.4
HJL-DDQ	6	34.41	45.21	50.31	54.12	58.91	64.5	67.5	72.69
ATZX-BTC	8	36.3	47.21	53.36	57.56	63.35	69.3	71.4	85.4
BJXZ-JSBWG	9	94.95	120.8	136.91	148.85	164.28	180.22	186.21	203.15
BTC-JDM	10	72.79	92.97	104.28	113.29	124.45	136.91	140.84	153.95
GM-JTXZ	10	81.25	102.59	116.62	125.88	138.62	151.56	156.48	172.35
XEQ-SD	13	152.30	208.23	223.16	247.18	262.46	281.34	290.47	348.22
